# Application of Primary/Secondary Circulating Tumor Cells for the Prediction of Biochemical Recurrence in Nonmetastatic Prostate Cancer Patients following Radical Prostatectomy or Radiotherapy: A Meta-Analysis

**DOI:** 10.1155/2021/4730970

**Published:** 2021-09-20

**Authors:** Liang Cao, Peng Hao, Dong Lin, Yangming Li, Tinghui Hu, Tao Cai, Shu Cui, Tao Wu

**Affiliations:** ^1^Department of Urology, Affiliated Hospital of North Sichuan Medical College, 1 Maoyuan South Road, Shunqing, Nanchong, 637000 Sichuan, China; ^2^Department of Urology, Pengzhou People's Hospital, 255 South Third Ring Road, Chengdu, 611900 Sichuan, China; ^3^Department of Urology, Emeishan People's Hospital, 2 Santaishan Fifth Street, Emeishan, 614200 Sichuan, China

## Abstract

**Background:**

Circulating tumor cells (CTCs) have been regarded as an independent prognostic marker for metastatic castration-resistant prostate cancer (mCRPC). Its prognostic value, however, in nonmetastatic prostate cancer (NMPC) is still unclear.

**Purpose:**

To elucidate whether CTCs can predict the biochemical recurrence (BCR) in NMPC patients following radical prostatectomy (RP) or radiotherapy (RT).

**Methods:**

PubMed, Cochrane Database, and Embase and the references in relevant studies were systematically searched. Studies that investigated the correlation of CTCs and BCR in NMPC patients after RP or RT were identified and reviewed. Overall odds ratio (OR) of BCR in such patients with/without CTCs was pooled. We also calculated and pooled overall prevalence of BCR in such CTC-positive patients.

**Results:**

In total, 12 studies comprising 1917 participants were eligible for the meta-analysis and showed that the presence of secondary circulating tumor cells (SCTCs) is associated with a higher BCR rate of 59% (95% CI: 22%-88%) in patients with NMPC after RP or RT (OR = 6.12; 95% CI: 2.22-16.85; *P* < 0.001). However, regardless of the presence of primary circulating tumor cells (PCTCs), it has not been shown to be associated with higher BCR.

**Conclusions:**

Our research demonstrated that SCTC-positive patients are associated with higher BCR compared to SCTC-negative patients in NMPC. Therefore, it is recommended that NMPC patients undergo CTC surveillance intensively after RP or RT.

## 1. Introduction

Both radical prostatectomy (RP) and radiotherapy (RT) are standard therapies for treating nonmetastatic prostate cancer (NMPC) [1]. Despite being considered a localized disease at the beginning of any anticancer therapy, 15-30% and 10-15% of patients will suffer biochemical recurrence (BCR) during the 5-year follow-up, respectively [2]. BCR is defined as detectable or rising prostate-specific antigen (PSA) value after surgery that is ≥0.2 ng/ml with a second confirmatory level of ≥0.2 ng/ml by the American Urological Association (AUA) [3]. Similarly, it is also defined as a rise in PSA to ≥2 ng/ml above nadir PSA after external beam radiotherapy (EBRT) with or without hormonal treatment by the American Society for Radiation Oncology (ASTRO) and Radiation Therapy Oncology Group (RTOG) [4]. Meanwhile, some studies have reported that BCR is associated with a significantly increased risk of metastasis and 24-34% of patients with BCR will develop into metastasis [5, 6]. Therefore, it is important to identify patients with high risk of treatment failure who should have benefited from early use of androgen deprivation therapy (ADT) or salvage radiotherapy (SRT) [7, 8]. Clinical stage, Gleason score (GS), and PSA have all already been established as independent prognostic factors for prostate cancer patients [9]. Also, PSA is the first and only one serum marker for the diagnosis of prostate cancer with a positive predictive value of 47% approved by the U.S. Food and Drug Administration since 1986; it seems to neither predict response to therapy nor present tumor progression [10–12].

Many studies have reported that the BCR of prostate cancer was predicted in different ways with related markers, including methods based on RNA, gene expression, protein expression, and genomic alteration. Nevertheless, the stratified predicted effect of these methods is not satisfactory. Therefore, new related markers will be explored to fill this field, especially the detection of circulating tumor cells (CTCs) which may help to predict the response to anticancer therapy and has recently attracted great attention. Ashworth firstly found that tumor cells were released into the peripheral circulation of patients in 1869 [13], commonly known as CTCs now. A subpopulation of tumor cells, also called primary circulating tumor cells (PCTCs), disseminates first to the neurovascular structures and then into the circulation, most of which are eliminated by host defenses or destroyed by shear forces [14, 15]. Though not all these cells are able to implant, PCTCs identify patients with possible clinically significant cancer. For example, those which express CD82, a tumor suppressor gene product linked to integration binding [16], are associated with upstaging and upgrading [17]. However, there may be a small part of PCTCs that will implant in distant tissues, survive, and in time proliferate. These cells will not be cleared by surgery and/or radiotherapy and may later be detected in the circulation which are called secondary circulating tumor cells (SCTCs) and thus represent minimal residual disease (MRD). CTC detection has been reported as a new noninvasive liquid-biopsy method applied in various solid tumors, such as breast cancer, pancreatic cancer, gastric cancer, and prostate cancer [18–21].

Recently, CTCs have been described as a reliable prognostic serum biomarker for metastatic castration-resistant prostate cancer (mCRPC) in many studies [22, 23]. However, some studies investigated the response of CTCs to RP or RT in NMPC showing different results [24, 25]. Consequently, we conducted a meta-analysis to investigate whether CTCs can predict the BCR in NMPC patients treated by RP or RT and to estimate the incidence rate of BCR in such CTC-positive patients.

## 2. Methods

The meta-analysis was conducted and reported according to the Preferred Reporting Items for Systematic Reviews and Meta-Analysis (PRISMA) statement [26], and the PRISMA checklist is detailed in the supplementary file (available here).

### 2.1. Data Sources and Search

A systematic literature search in PubMed, Cochrane Database, and Embase was conducted to identify relevant studies which investigated the correlation of CTCs and BCR in patients with NMPC after RP or RT published through January 2021. The search strategy was based on the combination of the following keywords: prostate neoplasm, prostate cancer, prostatic neoplasm, prostatic cancer; prostatectomy, radical prostatectomy; radiotherapy, radiation therapy, radiation treatment, targeted radiotherapy; biochemical relapse, biochemical recurrence. The reference list was also checked to retrieve other papers related to our topic. Any disagreements between the two authors (LC and PH) were settled by detailed discussion with a third investigator.

### 2.2. Study Selection

Two reviewers (LC and PH) independently reviewed the full texts of the potential eligible studies which met the following inclusion criteria: (1) the included subjects were NMPC patients treated by RP or RT and no evidence of BCR found when samples were collected; (2) the risk point estimate was reported as an odds ratio (OR) with the 95% CI, or the data were presented such that OR and 95% CI could be calculated; (3) the samples used in these studies should be peripheral blood; (4) the language in which the articles are compiled should be English; and (5) conference abstracts, review articles, and meta-analysis will be excluded. Studies that did not meet the earlier criteria were excluded.

### 2.3. Data Extraction and Quality Assessment

Two reviewers independently extracted following data from all eligible studies: first author's name, year of publication, number of cases and controls, study design, time of blood collection (before or after any treatment), treatments used in each study, blood sample volume, and methods of enrichment and detection of CTCs. BCR was defined as patients who have undergone primary treatment with prostatectomy or radiation, with rise to ≥0.2 from a prior undetectable level for prior prostatectomy or >2 mg/dl rise from postnadir radiotherapy.

Because the risk of bias in nonrandomized studies of interventions (ROBINS-I) quality assessment tool is not suitable for the quality evaluation of observational cohort studies, the Newcastle-Ottawa Scale (NOS) is used to define the methodological quality of each study [27]. A score of 6-9 is defined as high methodological quality, whereas a score less than 6 is low quality. NOS quality scores are presented as part of descriptive summaries for each study and did not influence decisions to pool studies in the meta-analysis.

### 2.4. Data Synthesis and Meta-Analysis

The synthesis of the odds ratio (OR) and the incidence rate of BCR was achieved by STATA ver14.0 (StataCorp, TX, USA) software and R ver4.0.4, respectively. Cochran's *Q* statistic and *I*^2^ statistic were used to assess the heterogeneity between all eligible studies. To explore studies which contribute to the heterogeneity of meta-analysis by omitting studies (leave one out at a time) from the meta-analysis, influence analysis would be performed if high heterogeneity (*I*^2^ > 50%) was found. Subgroup analysis was also used to explore the sources of heterogeneity. Similarly, the funnel chart was also used to assess publication bias. A 2-sided *P* < 0.05 was considered statistically significant for all statistical analysis.

## 3. Results

### 3.1. Literature Search and Study Selection

**Figure 1:**
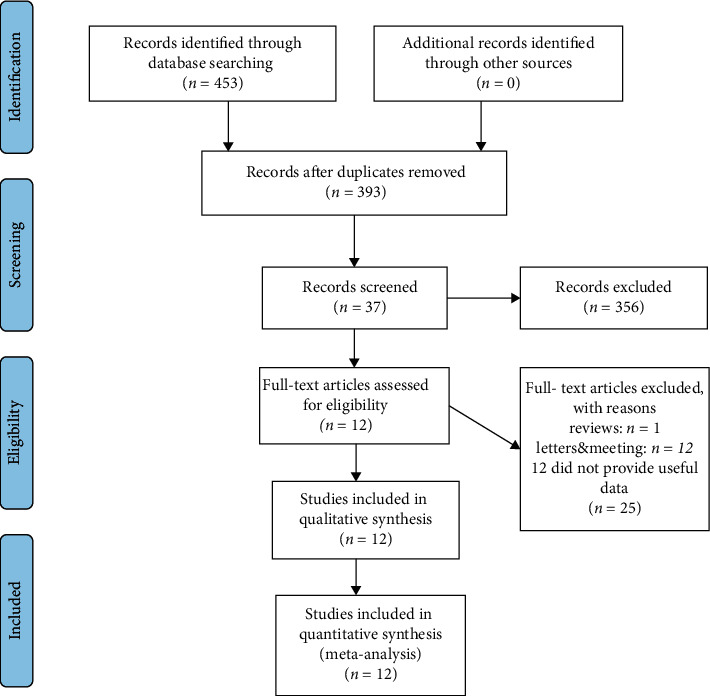
Flowchart for record selection process of the meta-analysis.

The flowchart of literature screening and selection results based on the PRISMA statement is shown in Figure 1. 453 records were obtained by a detailed search of the electronic database according to our prespecified search strategy, and 37 were considered potentially suitable. A total of 12 studies with 1917 participants were considered eligible for inclusion in the final meta-analysis after full-text review [16, 17, 24, 25, 28–35], and general characteristics of all included studies are shown in Table 1. Of the 12 studies, 9 explored the relationship between CTCs and BCR in NMPC patients who would undergo RP or RT [17, 24, 25, 28, 29, 32–35], and 8 investigated such a correlation in NMPC patients who had undergone RP or RT without evidence of BCR [16, 28, 30–35]. Meanwhile, 5 studies simultaneously discussed the relationship between the existence of CTCs before and after any anticancer therapies and BCR [28, 32–35].

### 3.2. Association between PCTCs and BCR

**Figure 2:**
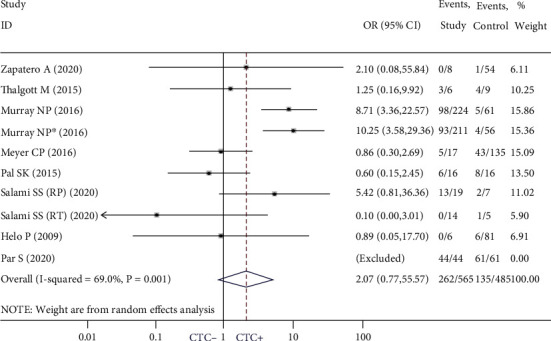
Pooled estimate of the association of primary circulating tumor cell-positive with biochemical recurrence.

We examined the relationship between PCTCs and BCR using data from 9 studies [17, 24, 25, 28, 29, 32–35]. There was no significant association between PCTCs and BCR (OR = 2.07; 95% CI: 0.77-5.57; *P* = 0.15; *I*^2^ = 69%) (Figure 2); the influence analysis showed the results were robust (Supplemental Figure 1). To explore the source of heterogeneity, we conducted a corresponding subgroup analysis; no significant relationship was found between PCTCs and BCR regardless of the treatment strategy (for RP [17, 24, 25, 28, 29, 32, 33, 35]: OR = 2.54; 95% CI: 0.90-7.15; *P* = 0.079; *I*^2^ = 72.6%) (for RT [25, 34]: OR = 0.48; 95% CI: 0.02-9.23; *P* = 0.625; *I*^2^ = 36.9%) (Supplemental Figure 2). There was also no significant association between PCTCs and BCR in studies using Cell Search System (CSS) to detect CTCs (OR = 0.87; 95% CI: 0.40-1.89; *P* = 0.725; *I*^2^ = 0.0%) [24, 32–34], whereas results from studies using other methods have shown a strong association between PCTCs and BCR (OR = 4.56; 95% CI: 1.52-13.65; *P* = 0.007; *I*^2^ = 53.5%) [17, 25, 28, 29, 35] (Supplemental Figure 3).

### 3.3. Association between SCTCs and BCR

**Figure 3:**
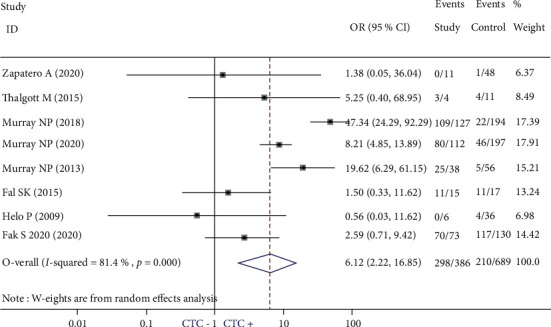
Pooled estimate of the association of secondary circulating tumor cell-positive with biochemical recurrence.

Eight studies focused on the relationship between SCTCs and BCR in NMPC patients treated by RP or RT [16, 28, 30–35], and a statistically significant association was found (OR = 6.12; 95% CI: 2.22-16.85; *P* < 0.001; *I*^2^ = 81.4%) (Figure 3). As showed in Supplemental Figure 4, our results were stable even after the sensitivity analysis was performed. Then, the subgroup analysis was performed to make an attempt to explain heterogeneity. We found that the presence of SCTCs was strongly associated with BCR regardless of the treatment regimen used (for RP [16, 28, 31–33, 35]: OR = 5.84; 95% CI: 1.36-25.08; *P* = 0.018; *I*^2^ = 84.9%) (for RT [30, 34]: OR = 7.17; 95% CI: 2.83-18.11; *P* < 0.001; *I*^2^ = 10.7%) (Supplemental Figure 5). Similarly, a statistically significant association was found between SCTCs and BCR in these studies that did not use CSS to detect CTCs (OR = 9.33; 95% CI: 2.93-29.74; *P* < 0.001; *I*^2^ = 85.7%) [16, 28, 30, 31, 35]. However, such a relationship has not been observed in these studies using CSS (OR = 1.96; 95% CI: 0.58-6.58; *P* = 0.278; *I*^2^ = 0.0%) [32–34] (Supplemental Figure 6).

### 3.4. BCR in CTC+ Patients

**Figure 4:**
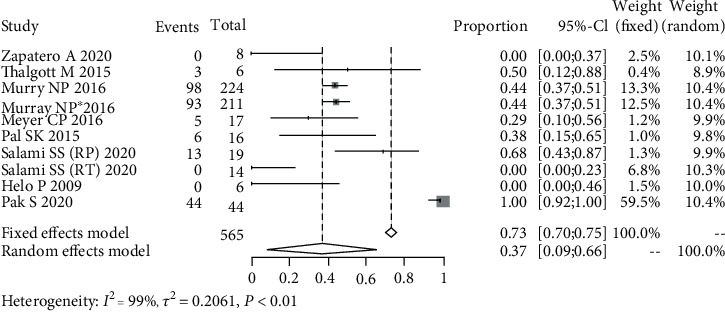
Pooled estimate of the incidence of biochemical recurrence in patients with primary circulating tumor cells.

**Figure 5:**
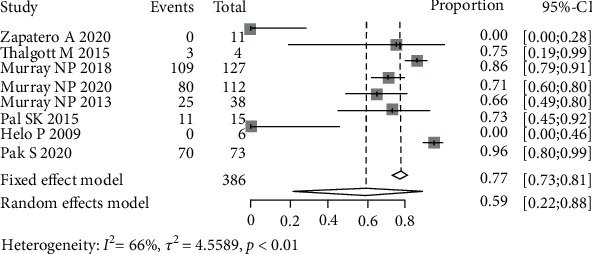
Pooled estimate of the incidence of biochemical recurrence in patients with secondary circulating tumor cells.

R software was used to integrate the incidence rate of BCR in primary or secondary CTC+ patients, and the incidence rate of BCR in primary and secondary CTC+ patients was 37% (95% CI: 9%-66%; *I*^2^ = 99%) (Figure 4) and 59% (95% CI: 22%-88%; *I*^2^ = 66%) (Figure 5), respectively. 65% of the BCR rate (95% CI: 56%-73%; *I*^2^ = 0%) (Supplemental Figure 7) was observed in these SCTC+ patients treated with RT [30, 34]. We also found BCR rates of 47% (95% CI: 19%-75%; *I*^2^ = 99%) [17, 24, 25, 28, 29, 32, 33, 35] (Supplemental Figure 8) and 73% (95% CI: 40%-92%; *I*^2^ = 69%) [16, 28, 31–33, 35] (Supplemental Figure 7) in patients who underwent RP with primary or secondary CTC+, respectively. Meanwhile, in studies with or without CSS, the cumulative incidence rate of BCR in PCTC+ patients was 26% (95% CI: 4%-49%; *I*^2^ = 76%) [24, 32–34] and 43% (95% CI: 7%-79%; *I*^2^ = 99%) [17, 25, 28, 29, 35], respectively (Supplemental Figure 9), while for SCTC+ patients, their incidence rate was 47% (95% CI: 30%-64%; *I*^2^ = 0%) [32–34] and 71% (95% CI: 32%-93%; *I*^2^ = 81%) [16, 28, 30, 31, 35], respectively (Supplemental Figure 10).

### 3.5. Publication Bias

Due to the limited number (below 10) of studies included in each meta-analysis, publication bias was not assessed.

## 4. Discussion

Meta-analysis of extractable data from 12 prospective studies demonstrated that the presence of SCTCs is associated with a higher BCR rate of 59% (95% CI: 22%-88%) in NMPC patients after RP or RT. However, despite the 37% of BCR rate, we found no statistical association between PCTCs and BCR in such patients.

Owing to the fact that only 3 studies have investigated the relationship between CTCs and BCR in patients undergoing RT, the present meta-analysis shows that the presence of SCTCs in such patients associated with a BCR rate of 65% remained to be verified. The relationship with PCTCs cannot be estimated due to the absence of positive events. Given the low predicted rate now, CTC testing may not be appropriate to predict BCR in patients undergoing RT. Therefore, more prospective studies are needed to further confirm this result.

For NMPC patients treated with RP, our results demonstrated that SCTCs are associated with a higher BCR rate of 73%. In other words, more than half of the patients that underwent RP will suffer from BCR once CTCs are detected. Chen et al. showed that CTCs in prostate cancer can be detected even without metastatic diseases and, therefore, that CTC monitoring has the potential to support early detection of disease progression in NMPC [36]. Meanwhile, a number of studies have demonstrated that early detection of cancer relapse or progression is associated with greater treatment options and better prognosis [37, 38]. Given such a high incidence rate, early intervention of ADT and/or SRT in these patients is possible, resulting in significant long-term prognostic benefits [8], and thus, excessive treatment could be avoided. Unfortunately, despite 37% of BCR rate, the association between PCTCs and BCR has not been found. In fact, two types of circulating prostate cells (CPCs) represent different clinical entities. SCTCs arising from a microfocus of micrometastatic diseases cannot be eliminated from the primary treatment and has great likelihood to lead to distant metastases compared with PCTCs that arise from the primary tumor, and not all of these PCTCs will survive or implant in distant tissues. Therefore, once the presence of SCTCs is detected, we should pay more attention to its indicative meaning and urge clinicians to intervene as early as possible.

One problem with CTC analysis is the variety of methods used to detect them, with varying degrees of specificity and sensitivity and the difference in their ability to detect malignant prostate cells rather than benign ones. Just as in benign colon diseases, CTCs which did not express P504S can be detected by CSS in men with benign prostate diseases [31, 39]. However, similar results and conclusions have been obtained in studies using different methods. Meyer et al. and Murray et al. both reported limited utility of PCTCs in predicting treatment outcome. Of the 12 included studies, 4 using CSS to detect CTCs demonstrated that no association between the presence of primary or secondary CTCs and BCR in NMPC patients after RP or RT was found. Meanwhile, a relatively poor BCR prediction rate was found in studies using the CSS method for PCTC+ (26%) or SCTCs+ (47%) patients, respectively. Actually, the number of CTCs detected by the CSS is underestimated in NMPC, which may be due to the fragmentation of traditional CTCs and disability to identify CTCs lacking epithelial characteristics [40]. At the same time, some studies have demonstrated that relying on epithelial cell adhesion molecule- (EPCAM-) based enrichment methods alone cannot completely detect all CTCs [41]. This may explain why the results of the data synthesis of studies using CSS did not show the correlation between primary/secondary CTCs and BCR. Of course, apart from the CSS, there are multiple methods to detect CTCs, including real-time polymerase chain reaction, cell size-based separation, or immunomagnetic beads conjugated with anti-EpCAM antibodies. The results obtained from studies that did not use the CSS show a great connection between primary/secondary CTCs and BCR in NMPC. Therefore, based on the current evidence, the conclusions drawn from studies using CSS need to be interpreted cautiously.

Up to now, CTCs have been regarded as an independent prognostic marker for mCRPC, but their prognostic value in NMPC is still unclear. This is the first meta-analysis to investigate whether the presence of CTCs is associated with a higher BCR rate in NMPC following RP or RT. In previous reports, a CTC detection rate of 5-27% was found in NMPC [42–44], and one thing that can be observed is that the detection rate of CTCs in NMPC is relatively low. Therefore, CTC+ would be clinically meaningful in predicting the long-term prognosis of these items, although our conclusions demonstrate that only the presence of SCTCs is associated with a higher BCR rate of 59% independent of the method used to detect them. Meanwhile, compared with the Walz nomogram, PCTCs did not predict BCR, whereas SCTCs did [17, 29]. In the 2019 European Urological Association Guideline, it is recommended that patients with BCR after RP undergo positron tomography computed tomography (PET-CT) examination, and for patients with BCR undergoing RT, multiparameter magnetic resonance imaging (MP-MRI) is recommended to localize abnormal areas and guide biopsies; also perform PET-CT with PSMA or fluciclovine or choline screening in these patients who are suitable for SRT [45]. Admittedly, the presence of CTCs in NMPC patients after RP or RT did not indicate whether tumor micrometastasis has occurred, but it can provide sufficient intervention intervals to reduce micrometastasis. Our results revealed that more than half of these SCTC+ patients would suffer from BCR. If these SCTC+ patients were provided routinely with the above screening only performed after BCR, will it detect early metastases while avoiding the problem of excessive examination? This is a question worth exploring and more research is needed to confirm it. Although the timing and treatment modality for PSA-only recurrences after RP or RT remain controversial based on the limited evidence, early SRT provides the possibility of cure for patients with an increasing PSA after RP. Boorjian et al. reported a 75% reduced risk of systemic progression with SRT, when comparing 856 SRT patients with 1801 non-SRT patients [46]. A retrospective analysis of 635 patients who were followed up after RP and experienced BCR and/or local recurrence and either received no salvage treatment (*n* = 397) or salvage RT alone (*n* = 160) within two years of BCR showed that salvage RT was associated with a threefold increase in prostate cancer-specific survival relative to those who received no salvage treatment [47]. Therefore, SRT is worth considering for NMPC patients following RP or RT with BCR, and it is worthy of cautious discussion to perform SRT especially for such SCTC+ patients. Thus, further studies, especially prospective studies, are required to advance knowledge in this field.

Several limitations of our meta-analysis are worth discussing. Although all eligible studies are prospective, selection bias cannot be avoided due to the fact that 5 of the eligible studies were done by the same team. At the same time, an article with a small sample size is included in this article, which may amplify the final conclusion.

## 5. Conclusions

Our research demonstrated that SCTC-positive patients are associated with higher BCR compared to SCTC-negative patients in NMPC. Therefore, it is recommended that NMPC patients undergo CTC surveillance intensively after RP or RT. Further studies, especially prospective studies, are required to better elucidate these relationships and to advance knowledge in this field.

## Figures and Tables

**Table 1:** Summary of eligible studies.

Study	Design	Country	Treatment procedures	CTC detection methods	Median (mean) initial PSA level (range) (ng/ml)	Median follow-up (range) (months)	Samples were collected prior to treatment		Samples were collected after treatment		NOS score (max: 9), for non-RCT
							Number of BF/CTC+ patients	Number of BF/CTC− patients	Number of BF/CTC+ patients	Number of BF/CTC− patients	
Zapatero et al., 2020 [34]	Prospective cohort study	Spain	RT	CSS	12.6 (3.2–68.7)	55 (10–40)	0/8	1/54	0/11	1/48	6
Thalgott et al., 2015 [33]	Prospective cohort study	Germany	RP	CSS	23 (40.7) (5.7–260.0)	44.3 (25–52)	3/6	4/9	3/4	4/11	6
Murray et al., 2016 [29]	Prospective cohort study	Chile	RP	Density gradient centrifugation+immunocytochemistry	5.89	60	98/224	5/61	—	—	7
Murray et al.,^∗^ 2016 [17]	Prospective cohort study	Chile	RP	Density gradient centrifugation+immunocytochemistry	5.7	60	93/211	4/56	—	—	7
Murray et al., 2018 [16]	Prospective cohort study	Chile	RP	Density gradient centrifugation+immunocytochemistry	5.52	(36–60)	—	—	109/127	22/194	7
Murray et al., 2020 [30]	Prospective cohort study	Chile	RT	Density gradient centrifugation+immunocytochemistry	6.3	96.36 (9.6–183.6)	—	—	80/112	46/197	8
Meyer et al., 2016 [24]	Prospective cohort study	Germany	RP	CSS	6.6 (0.04–87)	44.3 (48/36–60)	5/17	43/135	—	—	7
Murray et al., 2013 [31]	Prospective cohort study	Chile	RP	Density gradient centrifugation+immunocytochemistry	NR	NR	—	—	25/38	5/56	6
Pal et al., 2015 [32]	Prospective cohort study	America	RP	CSS	NR	17 (3.6–29.8)	6/16	8/16	11/15	11/17	7
Salami et al., 2019 [25]	Prospective cohort study	America	RP or RT	RBC lysis+immunocytochemistry	RP: 6.7 (1.8, 66.0) RT: 11.9 (1.2, 74.1)	14.2 (0.5–43.7)	RP: 13/19 RT: 0/14	RP: 2/7 RT: 1/5	—	—	7
Helo et al., 2009 [28]	Prospective cohort study	America	RP	RT-PCR	NR	NR	0/6	6/81	0/6	4/36	6
Pak et al., 2020 [35]	Prospective cohort study	Korea	RP	Based on replication-competent adenovirus	NR	(0–48)	44/44	61/61	70/73	117/130	8

RP: radical prostatectomy; RT: radiotherapy; CTC: circulating tumor cell; CSS: Cell Search System; RT-PCR: reverse transcription-polymerase chain reaction; RBC: red blood cell; BF: biochemical recurrence; PSA: prostate-specific antigen; NR: not reported.

## Data Availability

The data used to support the findings of this study are included within the article.

## Supplementary Materials

Supplementary Materials Supplemental Figure 1: influence analysis of meta-analysis of relationship between primary circulating tumor cell-positive and biochemical recurrence. Supplemental Figure 2: pooled estimate of subgroup analysis in the association of primary circulating tumor cell-positive with biochemical recurrence: radical prostatectomy vs. radiotherapy. Supplemental Figure 3: pooled estimate of subgroup analysis in the association of primary circulating tumor cell-positive with biochemical recurrence: Cell Search System vs. other tools. Supplemental Figure 4: influence analysis of meta-analysis of relationship between secondary circulating tumor cell-positive with biochemical recurrence. Supplemental Figure 5: pooled estimate of subgroup analysis in the association of secondary circulating tumor cell-positive with biochemical recurrence: radical prostatectomy vs. radiotherapy. Supplemental Figure 6: pooled estimate of subgroup analysis in the association of secondary circulating tumor cell-positive with biochemical recurrence: Cell Search System vs. other tools. Supplemental Figure 7: pooled estimate of subgroup analysis in the incidence of biochemical recurrence in patients with secondary circulating tumor cells: radical prostatectomy vs. radiotherapy. Supplemental Figure 8: pooled estimate of subgroup analysis in the incidence of biochemical recurrence in patients with primary circulating tumor cells: radical prostatectomy vs. radiotherapy. Supplemental Figure 9: pooled estimate of subgroup analysis in the incidence of biochemical recurrence in patients with primary circulating tumor cells: Cell Search System vs. other tools. Supplemental Figure 10: pooled estimate of subgroup analysis in the incidence of biochemical recurrence in patients with secondary circulating tumor cells: Cell Search System vs. other tools. We have provided a list of PRISMA-P; see file PRISMA-P-Checklist.docx for details.
